# Melatonin Protects against the Side-Effects of 5-Fluorouracil on Hippocampal Neurogenesis and Ameliorates Antioxidant Activity in an Adult Rat Hippocampus and Prefrontal Cortex

**DOI:** 10.3390/antiox10040615

**Published:** 2021-04-16

**Authors:** Kornrawee Suwannakot, Nataya Sritawan, Ram Prajit, Anusara Aranarochana, Apiwat Sirichoat, Wanassanun Pannangrong, Peter Wigmore, Jariya Umka Welbat

**Affiliations:** 1 Department of Anatomy, Faculty of Medicine, Khon Kaen University, Khon Kaen 40002, Thailand; kornrawee.s@kkumail.com (K.S.); nataya117@hotmail.com (N.S.); ram.prajit@kkumail.com (R.P.); anusar@kku.ac.th (A.A.); apiwsi@kku.ac.th (A.S.); wankun@kku.ac.th (W.P.); 2 Neurogenesis Research Group, Department of Anatomy, Faculty of Medicine, Khon Kaen University, Khon Kaen 40002, Thailand; 3 Queen’s Medical Centre, School of Life Sciences, Medical School, University of Nottingham, Nottingham NG7 2RD, UK; peter.wigmore@nottingham.ac.uk

**Keywords:** melatonin, 5-fluorouracil, hippocampus, prefrontal cortex, neurogenesis, antioxidant enzymes, oxidative stress, rat

## Abstract

Melatonin is an endogenous hormone that exhibits antioxidant functions and neuroprotective effects. The hippocampus and the prefrontal cortex (PFC) play an important role linked to working memory. 5-fluorouracil (5-FU) can induce oxidative stress and reduce neurogenesis in the subgranular zone (SGZ) of the dentate gyrus in a rat hippocampus and these alterations are related to working memory deficits. This study aimed to determine the effect of melatonin on 5-FU-induced oxidative stress that interferes with the antioxidant enzymes and protein expression levels in a rat hippocampus and PFC. A total of 68 male Sprague Dawley rats were divided into four groups: vehicle, 5-FU, melatonin and melatonin+5-FU groups. Rats were administered 5-FU (25 mg/kg, i.v.) on days 9, 12, 15, 18 and 21 and received melatonin (8 mg/kg, i.p.) at 19:00 from day 1 to day 21 of the experiment. Lipid peroxidation was assessed by measuring malondialdehyde (MDA) levels. Antioxidant enzyme levels including glutathione peroxidase (GPX), catalase (CAT) and superoxide dismutase (SOD) were determined. p21 immunofluorescence staining and Western blotting were used to detect the cell cycle arrest and protein expression of the nuclear factor erythroid 2-related factor 2 (Nrf2), doublecortin (DCX) and brain derived neurotrophic factor (BDNF), respectively. The results showed that melatonin reduced the number of p21-positive cells in the SGZ of the dentate gyrus and increased Nrf2, DCX and BDNF protein expression in rats treated with 5-FU. Moreover, melatonin restored antioxidant enzyme levels and reduced oxidative stress in the hippocampus and PFC caused by 5-FU. These findings reveal a mechanism of the neuroprotective properties of melatonin against 5-FU in a rat hippocampus and PFC.

## 1. Introduction

The hippocampus and prefrontal cortex (PFC) are brain regions associated with memory [1]. In the hippocampus, the tri-synaptic circuit plays an important role in memory formation especially spatial memory [2,3]. The PFC is important for recognition memory [4,5]. Several reports have found that cognitive impairment can be related to lesions and degeneration of neurons in the hippocampus and PFC [6,7,8,9]. Moreover, the reduction of neurogenesis can affect memory formation in the brain [10,11].

There are many factors that reduce neurogenesis such as receiving chemotherapy drugs [12,13,14]. 5-fluorouracil (5-FU), a chemotherapy drug, is one of the applied regimens used to treat cancer patients [15]. 5-FU induces its anticancer effects through the inhibition of thymidylate synthase (TS), which causes DNA damage [16,17]. Moreover, an active metabolite of 5-FU, fluorouridine triphosphate (FUTP), incorporates into RNA and disrupts normal RNA functions resulting in RNA damage [17,18]. Previous studies have revealed that the administration of 5-FU can cause cognitive impairment that is associated with decreasing cell proliferation, cell survival and the presence of immature neurons in the SGZ of the dentate gyrus in the adult rat hippocampus [11,19,20]. 5-FU activates oxidative stress by increasing intracellular ROS, which induces lipid peroxidation [21,22]. Moreover, 5-FU decreases the expression of doublecortin (DCX) and Nrf2 in rat hippocampi, which are commonly used as markers of immature neurons and the cellular antioxidant defense system, respectively [21].

Melatonin is chemically known as N-acetyl-5-methoxytryptamine and it has an intrinsic free radical scavenging capacity [23,24]. Previous studies have shown that melatonin ameliorates hippocampus-dependent spatial memory deficits in chemotherapy-treated rats [10,11]. These studies have demonstrated that melatonin does this by inducing neurogenesis by increasing cell proliferation, cell survival and the number of immature neurons in the SGZ of the dentate gyrus in the hippocampus [7,25,26]. Moreover, the regulation of neurogenesis is determined by neurotrophic factors such as BDNF [27]. A previous study has reported that melatonin can improve the BDNF expression in a rat vascular dementia model [28]. It is known that melatonin reduces oxidative stress via the stimulation of the antioxidant enzymes GPX, CAT and SOD [29,30]. Previously, melatonin was shown to increase glutathione (GSH) and SOD activities and decrease MDA levels in the hippocampus of developing rat pups [31].

The present study investigated the effect of melatonin on the adverse effects of 5-FU including the neuroprotective effects of melatonin on hippocampal neurogenesis, the modulation of GPX, CAT and SOD antioxidant enzyme activities and the protein expression levels of Nrf2, DCX and BDNF in a rat hippocampus and PFC.

## 2. Materials and Methods

### 2.1. Animals

The experimental protocols were approved by the Khon Kaen University Ethics Committee in Animal Research (Record No. IACUC-KKU-2/62, date of approval. 17 January 2019). Adult male Sprague Dawley rats (n = 7 animals/group) weighing 180–200 g were provided by Nomura Siam International in Thailand. The adult male Sprague Dawley rats were housed in a stainless cage with a 12 h light/dark cycle (06:00 to 18:00) with ad libitum food and water at room temperature 23 ± 2 °C.

### 2.2. Experimental Protocols

**Figure 1:**
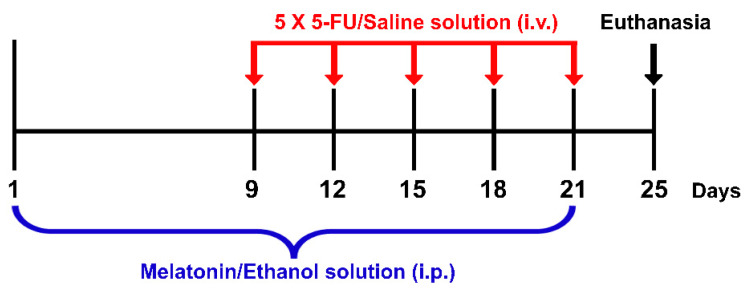
Timeline of drug administration. Red arrows represent each single intravenous (i.v.) injection of a 5-FU/Saline solution. The blue bracket represents the period of time for intraperitoneal (i.p.) injections of a melatonin/ethanol solution. Animals were killed and the brains were removed on day 25 as shown by the black arrow.

Melatonin and 5-FU were purchased from Sigma-Aldrich, St. Louis, MO, USA and Boryung Pharmaceutical Co., Ltd., Gyeonggi-do, Korea, respectively. Melatonin (8 mg/kg) was dissolved in 10% ethanol (RCI Labscan, Bangkok, Thailand) just before administration. Ethanol (10%) has been used to dissolve melatonin without any toxicity as described in several studies [7,11,32,33]. Animals were randomly assigned into four groups: vehicle, 5-FU, melatonin and melatonin+5-FU. Animals in the melatonin+5-FU group were administered with melatonin (8 mg/kg) by intraperitoneal (i.p.) injection once a day at 19:00 for 21 days from day 1 to day 21 and received a single dose of 5-FU (25 mg/kg) by intravenous (i.v.) injection at 10:00 on days 9, 12, 15, 18 and 21 of the experiment. The vehicle group was administered with an ethanol solution (10% ethanol dissolved in 0.9% NaCl at a volume of 1 mL/kg) by i.p. injection once a day at 19:00 for 21 days from day 1 to day 21 and received a single dose of a normal saline solution (0.9% NaCl, Thai Nakorn Patana Co., Ltd., Nonthaburi, Thailand) by i.v. injection at 10:00 on day 9, 12, 15, 18 and 21 of the experiment (Figure 1).

### 2.3. Tissue Sample Preparation

Animals were euthanized and decapitated. Brains from each animal were immediately harvested and one hemisphere was cryoprotected in a 30% sucrose solution (Ajax Finechem, Auckland, New Zealand) for 3 h at 4 °C until the brains sank to the bottom of the tubes [14]. The brains were embedded in an optimal cutting temperature (OCT) compound (Sakura Finetek, Torrance, CA, USA). After that, the embedded brains were frozen immediately in liquid nitrogen cooled isopentane (Sigma-Aldrich, St. Louis, MO, USA) and stored at −80 °C for p21 immunofluorescence staining [21]. The hippocampus and PFC were removed and snap-frozen in liquid nitrogen and stored at −80 °C for Western blotting and the determination of lipid peroxidation and antioxidant enzyme levels [6].

### 2.4. Biochemical Analysis

The hippocampus and prefrontal cortex were homogenized on ice to produce a 100 mg/mL solution using cold deionized water and then centrifuged at 13,000 rpm, 4 °C for 10 min. After that, the supernatant was carefully collected [6].

#### 2.4.1. Measurement of Malondialdehyde (MDA)

MDA levels were measured to analyze lipid peroxidation according to the protocols reported previously [6,21]. TEP or 1,1,3,3-Tetraethoxypropane (Sigma-Aldrich, St. Louis, MO, USA) was used as a standard solution. The supernatant of the hippocampus or PFC was mixed with 8.1% sodium dodecyl sulfate (Loba Chemie, Mumbai, India), 20% acetic acid (RCI Labscan, Bangkok, Thailand) and 0.8% thiobarbituric acid solutions (Sigma-Aldrich, St. Louis, MO, USA). The mixture was heated at 95 °C in a boiling water bath for 60 min and n-Butanol (RCI Labscan, Bangkok, Thailand) and pyridine (Loba Chemie, Mumbai, India) were added to the mixture and then vortexed. The mixture was centrifuged at 4000 rpm at 25 °C for 10 min. The optical density (OD) was read at 540 nm.

#### 2.4.2. Measurement of Catalase (CAT) Levels

CAT levels were evaluated using the method described previously [6]. A CAT enzyme solution (1000 units/mL, Sigma-Aldrich, St. Louis, MO, USA) was used as a standard solution. The supernatant was mixed with sulfuric acid (RCI Labscan, Bangkok, Thailand), 30% hydrogen peroxide (Merck, Darmstadt, Germany) and 50 mM potassium phosphate buffer, pH 7.0 (KH_2_PO_4_, Ajax Finechem, Auckland, New Zealand). The mixture was vortexed and incubated in the dark at room temperature for 10 min. The mixture was gently mixed with potassium permanganate solution, pH 7.0 (Ajax Finechem, Auckland, New Zealand). The OD was read at 540 nm.

#### 2.4.3. Measurement of Glutathione Peroxidase (GPX) Levels

GPX levels were carried out as described previously [6]. A GPX enzyme solution (20 units/mL, Sigma-Aldrich, St. Louis, MO, USA) was used as a standard solution. The supernatant was mixed with a reaction cocktail (pH 7.0) and DTNB or 5,5′-Dithiobis-(2-nitrobenzoic acid) (Sigma-Aldrich, St. Louis, MO, USA). The reaction cocktail contained a sodium phosphate buffer, pH 7.0 (NaH_2_PO_4_, Qrec, New Zealand), sodium azide (Sigma-Aldrich, St. Louis, MO, USA), L-glutathione oxidized (Sigma-Aldrich, St. Louis, MO, USA), β-NADPH or β-nicotinamide adenine dinucleotide phosphate (Sigma-Aldrich, St. Louis, MO, USA) and glutathione reductase solutions (Sigma-Aldrich, St. Louis, MO, USA). The mixture was vortexed and incubated in the dark at room temperature for 10 min and a 30% hydrogen peroxide solution was added to the mixture and then vortexed. The OD was read at 405 nm.

#### 2.4.4. Measurement of Superoxide Dismutase (SOD) Levels

SOD levels were examined as described previously [6]. A SOD enzyme solution (100 units/mL, Sigma-Aldrich, St. Louis, MO, USA) was used as a standard solution. The supernatant was mixed with a reaction cocktail (pH 7.8), which contained a 216 mM potassium phosphate buffer, pH 7.8 (K_2_HPO_4_, Ajax Finechem, Auckland, New Zealand), 10.7 mM ethylenediaminetatraacetic acid or EDTA (Sigma-Aldrich, St. Louis, MO, USA), 1.1 mM cytochrome C (Sigma-Aldrich, St. Louis, MO, USA) and 0.108 mM xanthine solutions (Sigma-Aldrich, St. Louis, MO, USA). A xanthine oxidase enzyme (XOD) solution (0.1 units/mL, Sigma-Aldrich, St. Louis, MO, USA) was added to the mixture and then vortexed. The OD was read at 540 nm and the absorbance was read at *t* = 0 min and *t* = 5 min.

### 2.5. Western Blot Analysis

Hippocampal and PFC tissue samples were homogenized on ice to produce a 100 mg/mL solution using a lysis buffer [34]. The samples were centrifuged at 13,000 rpm, 4 °C for 10 min and the supernatant was collected [6]. Protein concentrations were measured using the Lowry method [20]. A 12% separating gel was used to assess DCX, Nrf2 and BDNF expression. The samples (30 µg proteins/lane) were separated with SDS-PAGE and the proteins were transferred onto nitrocellulose membranes (GE Healthcare Life Sciences, Freiburg, Germany). The membranes were incubated with primary antibodies: anti-DCX (1:200, Santa Cruz Biotechnology, Dallas, TX, USA), anti-Nrf2 (1:1000, Abcam, Cambridge, UK), anti-BDNF (1:1000, Abcam, Cambridge, UK) and anti-GAPDH (1:20,000, Abcam, Cambridge, UK) overnight at 4 °C. After that, the membranes were incubated with secondary antibodies; anti-rabbit (1:2000, DAKO, Glostrup, Denmark), anti-goat (1:2000, DAKO, Glostrup, Denmark) and anti-mouse (1:2000, DAKO, Glostrup, Denmark) at room temperature for 60 min. An ECL (GE Healthcare, Buckingham, UK) Western blot detection system was used to visualize the protein bands. The images of protein bands were quantified for relative optical density using ImageJ software (version 1.53a, Wayne Rasband, National Institutes of Health, USA). Protein bands were detected according to the size of the proteins as follows: 68 kDa (Nrf2), 45 kDa (DCX), 36 kDa (GAPDH) and 14 kDa (BDNF). The relative optical density percentage of interested protein bands was compared with the internal control (GAPDH).

### 2.6. Immunofluorescence Study

Frozen brains were serially sectioned in a coronal plane covering the entire length of the dentate gyrus (bregma point −2.3 to −6.3 mm) according to the coordinate Atlas [35] using a cryostat (Cryostat Series HM550 Microm international, A.S. Science Co., Ltd., Walldorf, Germany). p21 immunofluorescence staining was performed to investigate the cell cycle arrest as described previously [6,21]. Forty micron sections were preserved in a cryoprotective buffer and stored in 24-well plates at 4 °C. Sections were incubated with an anti-p21 antibody (1:100, Santa Cruz Biotechnology, Texas, USA) at 4 °C overnight. Sections were incubated with an Alexa Fluor 488 rabbit anti-mouse IgG secondary antibody (1:300 Invitrogen, San Diego, CA, USA) at room temperature for 60 min and counterstained with propidium iodide (PI) (1:6000, Sigma-Aldrich, St. Louis, MO, USA). A systematic random sampling method was employed to choose every 8th section throughout the length of the dentate gyrus [36]. Nine sections were used for staining using a free floating method. p21-positive cells within three cells of the inner edge in the upper and lower blade of the DG were counted [10,11,37]. Sections were viewed at 10 × and 40 × by a Nikon ECLIPSE 80i fluorescence microscope (Melville, NY, USA).

### 2.7. Statistical Analysis

The statistical parameters were calculated using GraphPad Prism (version 5.0; GraphPad software Inc., San Diego, CA, USA). Statistically significant differences were considered at probability levels of *p* < 0.05, *p* < 0.01 and *p* < 0.001. A one-way analysis of variance (ANOVA) was used to analyze the data of immunofluorescence, Western blotting, lipid peroxidation measurement and antioxidant enzyme activity. A Bonferroni post-hoc test was performed when the ANOVA was significant. All of the data are expressed as a mean ± SEM.

## 3. Results

### 3.1. Melatonin Decreased the Expression of p21 and Prevented Cell Cycle Arrest Caused by 5-FU

**Figure 2:**
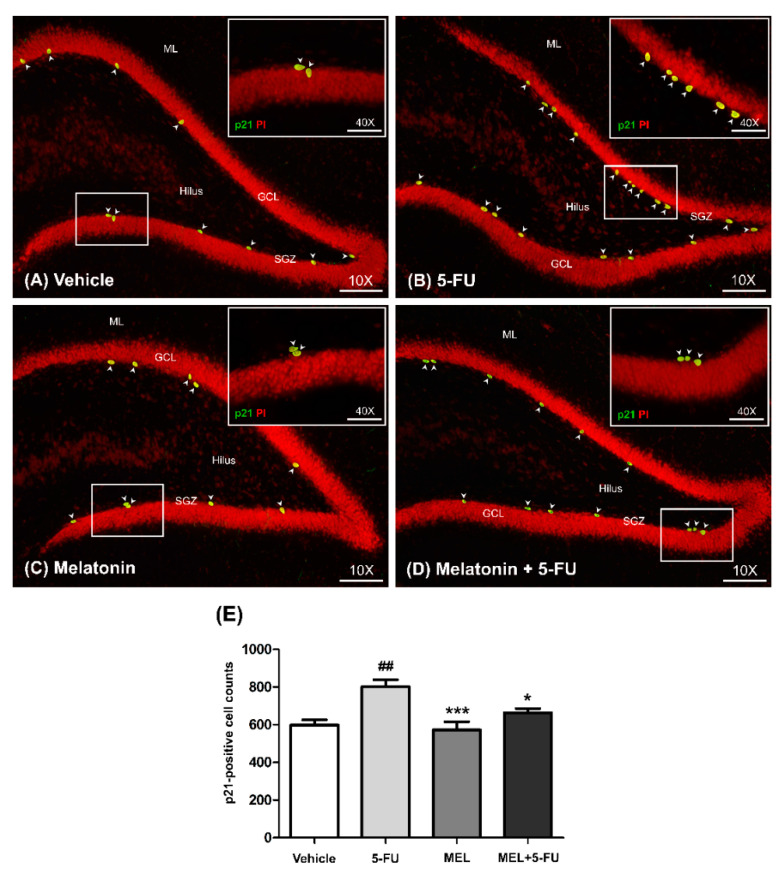
Immunofluorescence staining of a cell cycle arrest. Green p21-positive cells are indicated with arrowheads in the SGZ of the dentate gyrus (**A**–**D**). Sections were counterstained with red nuclear dye, propidium iodide (PI). Inserted images show p21 immunofluorescence staining under high magnification (Bar scales: 50 μm, 40×). The number of p21-positive cells in the 5-FU group was significantly higher than those in the vehicle group (**E**). ## *p* < 0.01 compared with the vehicle group. * *p* < 0.05, *** *p* < 0.001 compared with the 5-FU group. Subgranular zone: SGZ; granule cell layer: GCL; molecular layer: ML.

p21 is commonly used as a marker of cell cycle arrest [38]. The number of p21-positive cells in the SGZ of the dentate gyrus in the hippocampus was determined using immunofluorescence staining. The number of p21-positive cells in the vehicle, melatonin and melatonin co-administration groups were significantly lower than the animals treated with 5-FU alone (mean ± SEM; vehicle: 598.70 ± 26.81 cells, 5-FU: 801.30 ± 37.15 cells, melatonin: 573.30 ± 42.11 cells, melatonin+5-FU: 662.70 ± 22.48 cells, *p* < 0.05, *p* < 0.01, *p* < 0.001, Figure 2A–E). However, there were no significant differences in p21-positive cells in the vehicle and melatonin groups (*p* < 0.05). This result suggested that melatonin could prevent 5-FU-induced cell cycle arrest in the SGZ of the hippocampal dentate gyrus.

### 3.2. Melatonin Restored Antioxidant Enzyme Levels and Reduced Lipid Peroxidation in the Hippocampus and PFC

#### 3.2.1. MDA Levels

**Figure 3:**
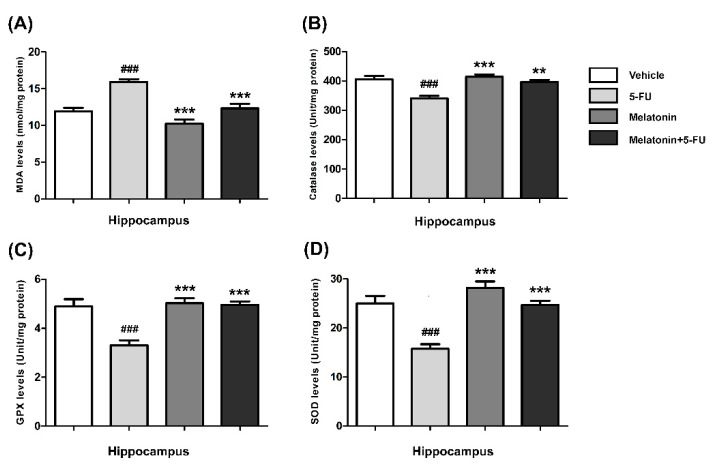
MDA (**A**), CAT (**B**), GPX (**C**) and SOD (**D**) levels in the hippocampus. ### *p* < 0.001 compared with the vehicle group. ** *p* < 0.01 and *** *p* < 0.001 compared with the 5-FU group. Malondialdehyde: MDA; catalase: CAT; glutathione peroxidase: GPX; superoxide dismutase: SOD.

**Figure 4:**
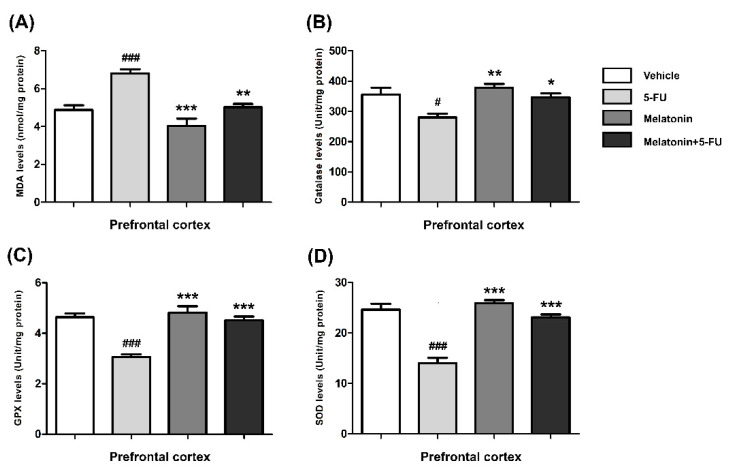
MDA (**A**), CAT (**B**), GPX (**C**) and SOD (**D**) levels in the PFC. # *p* < 0.05 and ### *p* < 0.001 compared with the vehicle group. * *p* < 0.05, ** *p* < 0.01 and *** *p* <0.001 compared with the 5-FU group. Malondialdehyde: MDA; catalase: CAT; glutathione peroxidase: GPX; superoxide dismutase: SOD.

The MDA levels in the hippocampus of animals treated with 5-FU alone were significantly higher than those in the vehicle, melatonin and melatonin+5-FU groups (mean ± SEM; vehicle: 11.91 ± 0.50 nmol/mg protein, 5-FU: 15.92 ± 0.37 nmol/mg protein, melatonin: 10.24 ± 0.57 nmol/mg protein, melatonin+5-FU: 12.32 ± 0.62 nmol/mg protein, *p* < 0.001, Figure 3A). In the PFC, the MDA levels of animals in the vehicle, melatonin and melatonin co-administration groups were significantly lower than the 5-FU-treated animals (mean ± SEM; vehicle: 4.87 ± 0.25 nmol/mg protein, 5-FU: 6.80 ± 0.23 nmol/mg protein, melatonin: 4.03 ± 0.39 nmol/mg protein, melatonin+5-FU: 5.03 ± 0.17 nmol/mg protein, *p* < 0.01, *p* < 0.001, Figure 4A). However, there were no significant differences in MDA levels in the hippocampus and PFC in the vehicle and melatonin groups (*p* < 0.05).

#### 3.2.2. Catalase (CAT) Levels

The CAT levels in the hippocampus of the melatonin and melatonin+5-FU groups were significantly higher than the 5-FU group but not significantly higher than the vehicle group (mean ± SEM; vehicle: 405.70 ± 11.44 unit/mg protein, 5-FU: 340.40 ± 8.64 unit/mg protein, melatonin: 414.00 ± 8.47 unit/mg protein, melatonin+5-FU: 396.80 ± 6.66 unit/mg protein, *p* < 0.01, *p* < 0.001, Figure 3B). The CAT levels in the PFC of animals treated with 5-FU alone were significantly decreased when compared with the vehicle, melatonin and melatonin+5-FU groups (mean ± SEM; 5-FU: 280.30 ± 11.59 unit/mg protein, vehicle: 356.00 ± 22.19 unit/mg protein, melatonin: 377.50 ± 13.15 unit/mg protein, melatonin+5-FU: 346.40 ± 12.91 unit/mg protein, *p* < 0.05, *p* < 0.01, Figure 4B). The CAT levels in the hippocampus and PFC were not significantly different between the vehicle and melatonin groups (*p* < 0.05).

#### 3.2.3. Glutathione Peroxidase (GPX) Levels

In the hippocampus, the GPX levels of animals in the melatonin and melatonin+5-FU groups were not significantly increased in comparison with the vehicle group but were significantly increased when compared with the 5-FU group (mean ± SEM; vehicle: 4.89 ± 0.30 unit/mg protein, 5-FU: 3.30 ± 0.21 unit/mg protein, melatonin: 5.02 ± 0.20 unit/mg protein, melatonin+5-FU: 4.95 ± 0.14 unit/mg protein, *p* < 0.001, Figure 3C). The levels of GPX in the PFC in the animals given 5-FU alone significantly differed from those in the vehicle, melatonin and melatonin+5-FU groups (mean ± SEM; vehicle: 4.64 ± 0.15 unit/mg protein, 5-FU: 3.06 ± 0.10 unit/mg protein, melatonin: 4.83 ± 0.25 unit/mg protein, melatonin+5-FU: 4.51 ± 0.15 unit/mg protein, *p* < 0.001, Figure 4C). However, there were no significant differences in the GPX levels in the hippocampus and PFC in the vehicle and melatonin groups (*p* < 0.05).

#### 3.2.4. Superoxide Dismutase (SOD) Levels

A significant reduction of the SOD levels in the hippocampus was found in the 5-FU group when compared with the vehicle, melatonin and melatonin co-administration groups (mean ± SEM; vehicle: 25.00 ± 1.54 unit/mg protein, 5-FU: 15.73 ± 0.95 unit/mg protein, melatonin: 28.16 ± 1.32 unit/mg protein, melatonin+5-FU: 24.68 ± 0.85 unit/mg protein, *p* < 0.001, Figure 3D). In the PFC, there were no differences in the SOD levels among the vehicle, melatonin and melatonin+5-FU groups; however, the SOD levels in these groups were significantly increased when compared with animals in the 5-FU group (mean ± SEM; vehicle: 24.65 ± 1.16 unit/mg protein, 5-FU: 14.04 ± 1.02 unit/mg protein, melatonin: 25.89 ± 0.62 unit/mg protein, melatonin+5-FU: 23.10 ± 0.59 unit/mg protein, *p* < 0.001, Figure 4D). The SOD levels in the hippocampus and PFC were not significantly different between the vehicle and melatonin groups (*p* < 0.05).

### 3.3. Melatonin Increased the Expression of Nrf2, DCX and BDNF in the Hippocampus and PFC

**Figure 5:**
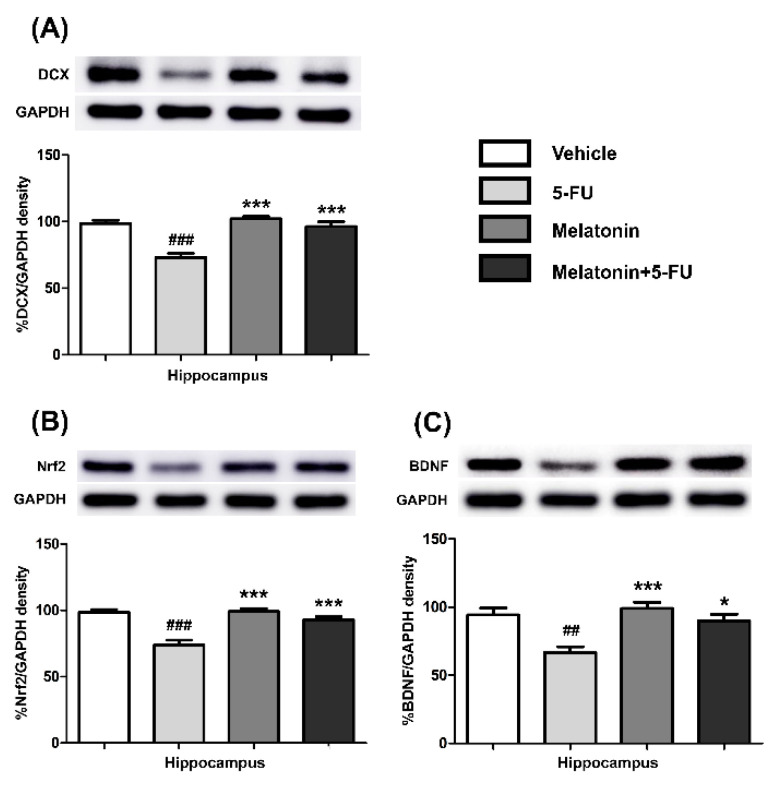
DCX (**A**), Nrf2 (**B**) and BDNF (**C**) protein expression in the hippocampus. ## *p* < 0.01 and ### *p* < 0.001 compared with the vehicle group. * *p* < 0.05 and *** *p* < 0.001 compared with the 5-FU group. Doublecortin: DCX; nuclear factor erythroid 2-related factor 2: Nrf2; brain derived neurotrophic factor: BDNF.

There were no differences in the expression of Nrf2, DCX and BDNF proteins in the hippocampus of the vehicle, melatonin and melatonin+5-FU groups, as shown in Figure 5A–C. Both the melatonin and melatonin+5-FU groups exhibited significant increases in the expression of DCX (mean ± SEM; 5-FU: 72.95 ± 3.21, melatonin: 102.10 ± 1.83, melatonin+5-FU: 96.34 ± 3.63, *p* < 0.001, Figure 5A), Nrf2 (mean ± SEM; 5-FU: 73.84 ± 3.71, melatonin: 99.21 ± 2.25, melatonin+5-FU: 92.93 ± 2.33, *p* < 0.001, Figure 5B) and BDNF (mean ± SEM; 5-FU: 66.77 ± 4.17, melatonin: 99.09 ± 4.51, melatonin+5-FU: 89.81 ± 4.96, *p* < 0.05, *p* < 0.01, *p* < 0.001, Figure 5C) in the hippocampus when compared with the 5-FU group. However, there were no significant differences in the expression of Nrf2, DCX and BDNF proteins in the hippocampus in the vehicle and melatonin groups (*h* 0.05).

**Figure 6:**
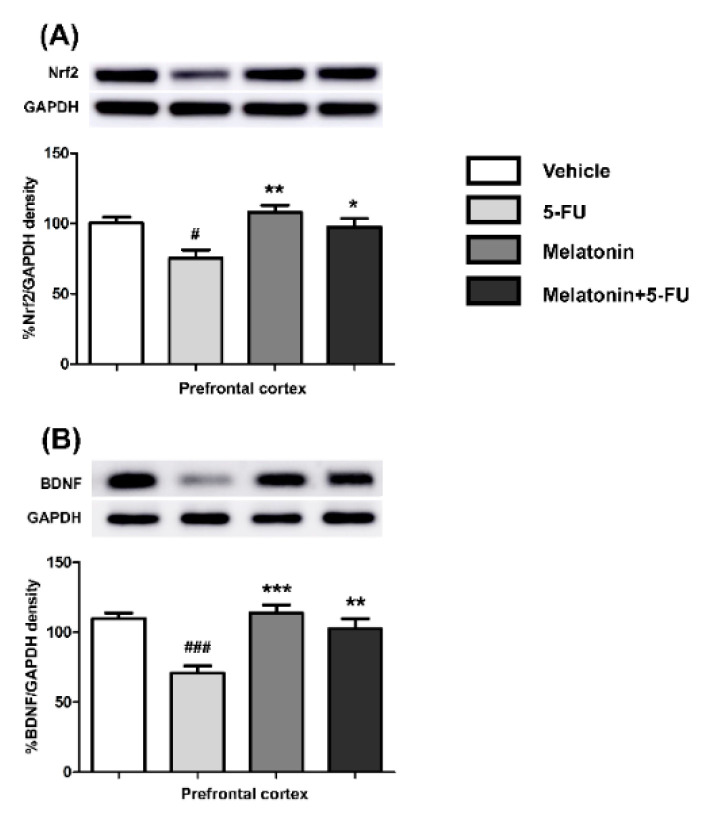
Nrf2 (**A**) and BDNF (**B**) protein expression in the PFC. # *p* < 0.05 and ### *p* < 0.001 compared with the vehicle group. * *p* < 0.05, ** *p* < 0.01 and *** *p* < 0.001 compared with the 5-FU group. Doublecortin: DCX; nuclear factor erythroid 2-related factor 2: Nrf2; brain derived neurotrophic factor: BDNF.

There were significant differences in the Nrf2 and BDNF expression levels in the PFC between the vehicle and the 5-FU groups as shown in Figure 6A,B. There were significant increases in the expression of Nrf2 (mean ± SEM; 5-FU: 75.32 ± 5.86, melatonin: 108.20 ± 4.87, melatonin+5-FU: 97.58 ± 5.95, *p* < 0.05, *p* < 0.01, Figure 6A) and BDNF (mean ± SEM; 5-FU: 70.80 ± 5.09, melatonin: 113.90 ± 5.73, melatonin+5-FU: 102.70 ± 6.93, *p* < 0.01, *p* < 0.001, Figure 6B) in the PFC in the melatonin and melatonin+5-FU groups when compared with the 5-FU group. The Nrf2 and BDNF expression levels in the PFC were not significantly different between the vehicle and melatonin groups (*p* < 0.05). These findings suggested that melatonin could restore the DCX, Nrf2 and BDNF expression to normal levels in animals that received 5-FU.

## 4. Discussion

In this study, 5-FU-treated rats showed an increase in the number of p21-positive cells in the SGZ of the dentate gyrus in the hippocampus, which was associated with a reduction in hippocampal neurogenesis via a decrease in the DCX protein expression in the hippocampal tissue. In addition, 5-FU increased the MDA levels and decreased the CAT, GPX and SOD antioxidant activities and reduced the expression of BDNF and Nrf2 in both the hippocampus and the PFC. Melatonin prevented 5-FU-induced cell cycle arrest and oxidative damage by improving antioxidant activity and restoring the expression of BDNF, Nrf2 and DCX to normal levels.

Neurogenesis occurs throughout life in the SGZ of the hippocampal dentate gyrus and the subventricular zone of the lateral ventricles. Our previous studies have demonstrated that animals that received 5-FU showed a marked decrease in neurogenesis [11,14]. 5-FU is an anticancer drug in the antimetabolite group that is used in several regimens to treat solid cancers of the gastrointestinal tract, breast, head and neck and pancreas [15]. The anticancer effects of 5-FU are mediated through induced DNA and RNA damage [17]. Animals that received 5-FU showed a dramatic decline in cell proliferation and cell survival in the SGZ of the hippocampal dentate gyrus and had reduced numbers of DCX-positive cells [11]. DCX is a microtubule-associated protein that plays an essential role in neuronal migration and acts as an immature neuronal marker [25,26]. This current study extended this observation and showed that the animals in the 5-FU group also had an increase in p21-positive cells in the SGZ of the hippocampal dentate gyrus [21]. p21 is a cyclin-dependent kinase inhibitor protein (CDI), which inhibits the cyclin-dependent kinases (CDKs) that play a key role in regulating the cell cycle [39]. An increase in p21 indicates an increase in cell cycle arrest [38]. Therefore, this study confirmed that 5-FU could enhance cell cycle arrest, causing a decrease in neurogenesis in the SGZ of the hippocampal dentate gyrus. In addition, this reduction in the neurogenesis caused by 5-FU correlated with a marked decrease in the DCX expression in the hippocampus. Our previous studies in rat models have shown that melatonin could improve chemotherapy and valproic acid (VPA)-induced impairment of neurogenesis including cell proliferation, the survival of new neurons and the number of immature neurons in the SGZ of the dentate gyrus in the hippocampus [7,10,11]. The present study found that rats in the melatonin and melatonin+5-FU groups showed a significant decrease in the number of p21-positive cells in the SGZ of the hippocampal dentate gyrus. This finding confirmed that the co-administration of melatonin with 5-FU could prevent cell cycle arrest, which ameliorated the reduction of the neurogenesis in the SGZ of the hippocampal dentate gyrus.

Reactive oxygen species (ROS) are free radicals containing oxygen centered radicals [40,41]. An excess of ROS causes biological damage that is termed oxidative stress [42]. Oxidative stress is the result of an imbalance between the generation of ROS and the antioxidant capability of the cells [43]. An earlier study showed that 5-FU induced oxidative stress by enhancing ROS generation resulting in a remarkable increase in MDA, which is the main product of lipid peroxidation [22]. The current study found that the animals in the 5-FU group produced higher MDA levels in the hippocampus and PFC when compared with the vehicle group. This result demonstrated that 5-FU could cause oxidative stress via inducing lipid peroxidation, which correlated with the increase in the number of p21-positive cells when compared with the vehicle group. It is known that p21 is triggered when ROS generation is increased and oxidative stress is induced in the cell [44]. Therefore, these results indicated that the 5-FU-induced cell cycle arrest seen in the SGZ of the hippocampal dentate gyrus was caused by an increase in oxidative stress. Moreover, the increase in oxidative stress activated by 5-FU was confirmed by the decline of the GPX, CAT and SOD levels in the hippocampus and PFC in this study.

In general, all living organisms maintain a balance between the generation and the destruction of ROS [45]. Three types of antioxidant enzymes (SOD, CAT and GPX) are important in the mechanism for destroying ROS [46]. The present study displayed significant increases of SOD, CAT and GPX levels in the hippocampus and PFC of animals treated with melatonin and melatonin+5-FU when compared with the 5-FU group. This finding showed that melatonin could induce increased antioxidant enzyme levels in both the hippocampus and PFC, which were important in the mechanism for destroying ROS in the cells after 5-FU treatment. Similarly, several studies have reported that melatonin has antioxidant properties and functions as a free radical scavenger [30,31,47]. Melatonin functions as a protector against ROS and reactive nitrogen species (RNS) [24,48]. Melatonin also indirectly reduces oxidative stress via the stimulation of antioxidant enzymes [29]. From the present study, moreover, the increase in antioxidant enzyme levels was associated with the increase in the Nrf2 expression in both the hippocampus and the PFC. Nrf2 plays a key role in regulating antioxidant production to maintain cellular redox homeostasis [49,50]. Nrf2 normally binds to kelch-like ECH-associated protein 1 (Keap1) in the cytoplasm [51]. Under conditions of oxidative stress, the interaction between Keap1 and Nrf2 is disrupted, which activates Nrf2 to be translocated into the nucleus and bound to the antioxidant response element (ARE) [52]. Afterwards, Nrf2 promotes the transcription of many antioxidant and detoxification genes, which include enzymes such as heme oxygenase-1 (HO-1), CAT, SOD, GPX and glutathione S-transferase (GST). These enzymes reduce oxidative stress and free radicals in cells [50]. Therefore, the Nrf2/ARE signaling pathway acts as an antioxidant defense system [53]. This study found that both the melatonin and melatonin+5-FU groups exhibited significant increases in the expression of Nrf2 in the hippocampus and the PFC when compared with the 5-FU group. Similarly, a previous study showed that the attenuating effect of melatonin on oxidative damage induced by isoflurane was related to the PKCα/Nrf2 signaling pathway in developing rats [31]. These results showed that the co-administration of melatonin with 5-FU efficiently enhanced Nrf2 levels in the hippocampus and the PFC, which played roles in the antioxidant defense system against the effect of 5-FU.

BDNF is one of the neurotrophin family of growth factors [54]. BDNF specifically interacts with tropomyosin receptor kinase B (TrkB) to activate the cAMP response element binding protein (CREB) transcription factor, which results in an increase of synaptic plasticity and neurogenesis linked to learning and memory [27,55,56,57]. A previous study has shown that 5-FU impairs newborn neurons and spatial working memory, a hippocampus-dependent memory that is associated with a decreased BDNF expression in the hippocampus [34]. In this study, 5-FU administration significantly suppressed the BDNF expression both in the hippocampus and PFC when compared with the vehicle group. The PFC regulates recognition memory [4]. Therefore, the reductions of BDNF levels in the hippocampus and PFC after 5-FU administration found in this study were likely to lead to impairments in both spatial and recognition memory in adult rats. Our previous studies have shown that melatonin can relieve spatial and recognition memory deficits in adult rats [7,10,11]. In addition, melatonin and resveratrol can improve the BDNF expression in the hippocampus of bilateral common carotid artery occlusion (BCCAO)-treated rats, resulting in an improvement in cognitive deficits as tested by the Morris water maze and novel object recognition tests [28]. This study showed that the BDNF expression in the melatonin+5-FU group was restored to normal levels in the hippocampus and PFC. These results demonstrated that the co-administration of melatonin in 5-FU-treated rats proficiently up-regulated the BDNF expression, which plays a critical role in synaptic plasticity, neurogenesis and memory in both the hippocampus and PFC.

## 5. Conclusions

In conclusion, this study shows a mechanism for the neuroprotective properties of melatonin against 5-FU in a rat hippocampus and PFC. Melatonin ameliorated the effects of 5-FU on hippocampal neurogenesis by decreasing the cell cycle arrest and increasing the DCX expression in the hippocampus. Furthermore, melatonin up-regulated the BDNF expression, reduced oxidative stress and stimulated antioxidant activity in the hippocampus and PFC. Therefore, these findings illustrate the neuroprotective properties of melatonin and provide an understanding of its mechanism to prevent the impairment of neurogenesis and memory deficits in patients who have received 5-FU.

## Data Availability

All data is contained within the article.

## Abbreviations

5-fluorouracil: 5-FU; antioxidant response element: ARE; bilateral common carotid artery occlusion: BCCAO; brain derived neurotrophic factor: BDNF; catalase: CAT; cyclin-dependent kinase inhibitor protein: CDI; cyclin-dependent kinases: CDKs; cAMP response element binding protein: CREB; doublecortin: DCX; fluorouridine triphosphate: FUTP; granule cell layer: GCL; glutathione peroxidase: GPX; glutathione: GSH; glutathione S-transferase: GST; heme oxygenase-1: HO-1; intraperitoneal: i.p.; intravenous: i.v.; kelch-like ECH-associated protein 1: Keap1; malondialdehyde: MDA; molecular layer: ML; nuclear factor erythroid 2-related factor 2: Nrf2; optimal cutting temperature: OCT; optical density: OD; prefrontal cortex: PFC; propidium iodide: PI; reactive nitrogen species: RNS; reactive oxygen species: ROS; subgranular zone: SGZ; superoxide dismutase: SOD; tropomyosin receptor kinase B: TrkB; thymidylate synthase: TS; valproic acid: VPA; xanthine oxidase: XOD.
